# Current Nomenclature of Pseudohypoparathyroidism: Inactivating Parathyroid Hormone/Parathyroid Hormone-Related Protein Signaling Disorder

**DOI:** 10.4274/jcrpe.2017.S006

**Published:** 2017-12-30

**Authors:** Serap Turan

**Affiliations:** 1 Marmara University Faculty of Medicine, Department of Pediatric Endocrinology, İstanbul, Turkey

**Keywords:** Pseudohypoparathyroidism, inactivating parathyroid hormone/parathyroid hormone related protein signaling disorder

## Abstract

Disorders related to parathyroid hormone (PTH) resistance and PTH signaling pathway impairment are historically classified under the term of pseudohypoparathyroidism (PHP). The disease was first described and named by Fuller Albright and colleagues in 1942. Albright hereditary osteodystrophy (AHO) is described as an associated clinical entity with PHP, characterized by brachydactyly, subcutaneous ossifications, round face, short stature and a stocky build. The classification of PHP is further divided into PHP-Ia, pseudo-PHP (pPHP), PHP-Ib, PHP-Ic and PHP-II according to the presence or absence of AHO, together with an in vivo response to exogenous PTH and the measurement of Gsα protein activity in peripheral erythrocyte membranes in vitro. However, PHP classification fails to differentiate all patients with different clinical and molecular findings for PHP subtypes and classification become more complicated with more recent molecular characterization and new forms having been identified. So far, new classifications have been established by the EuroPHP network to cover all disorders of the PTH receptor and its signaling pathway. Inactivating PTH/PTH-related protein signaling disorder (iPPSD) is the new name proposed for a group of these disorders and which can be further divided into subtypes - iPPSD1 to iPPSD6. These are termed, starting from PTH receptor inactivation mutation (Eiken and Blomstrand dysplasia) as iPPSD1, inactivating Gsα mutations (PHP-Ia, PHP-Ic and pPHP) as iPPSD2, loss of methylation of GNAS DMRs (PHP-Ib) as iPPSD3, PRKAR1A mutations (acrodysostosis type 1) as iPPSD4, PDE4D mutations (acrodysostosis type 2) as iPPSD5 and PDE3A mutations (autosomal dominant hypertension with brachydactyly) as iPPSD6. iPPSDx is reserved for unknown molecular defects and iPPSDn+1 for new molecular defects which are yet to be described. With these new classifications, the aim is to clarify the borders of each different subtype of disease and make the classification according to molecular pathology. The iPPSD group is designed to be expandable and new classifications will readily fit into it as necessary.

## INTRODUCTION

Pseudohypoparathyroidism (PHP) is a group of rare, related, highly heterogeneous disorders, which are characterized by end-organ resistance to parathyroid hormone (PTH) action. PHP and related disorders are caused by the genetic and/or epigenetic changes leading to down-regulation of a cyclic adenosine monophosphate (cAMP) generator, mostly related to the GNAS gene (1,2,3,4,5). GNAS is an imprinted gene which gives rise to multiple gene products, including transcripts that encode the α*-subunit of the stimulatory guanine nucleotide-binding protein (G protein) (Gsα), extra-large Gsα (XLαs), and neuroendocrine secretory protein* 55 (NESP55), as well as to noncoding A/B (also referred to as 1A) and *antisense transcripts* (GNAS-AS1).

Gsα is a ubiquitously expressed signaling protein having a role in the actions of many hormones and other endogenous molecules through the generation of intracellular cAMP and encoded by GNAS exons 1-13 (1,2,3,4,5). Other GNAS transcripts NESP55, XLαs, and A/B, with the exception of GNAS-AS1 consists of distinct exons, and all contain their own, differentially methylated, unique first exons (DMRs), which are spliced onto exon 2 of GNAS. So all of these transcripts, from exon 2 on, are identical in sequence to Gsα (6,7,8,9,10,11). Thus a structural or epigenetic change in other GNAS trancripts also affects Gsα function.

Expression patterns of Gsα and other GNAS transcripts in different tissues determine the disease phenotype when GNAS mutations are present. The Gsα transcript is biallelically expressed in most tissues. However, silenced paternal Gsα expression in some tissues, including proximal renal tubules, neonatal brown adipose tissue, thyroid, gonads, the paraventricular nucleus of the hypothalamus and pituitary can cause hormone resistance in cases of maternal mutations (12,13,14,15,16,17,18). Thus, mutations on maternal alleles cause hormone resistance i.e. PHP.

Historically PHP is the first hormone-resistance syndrome, described by Albright et al (19) and characterized by hypocalcemia, hyperphosphatemia, and elevated PTH levels and Albright hereditary osteodystrophy (AHO). Clinical features of AHO are obesity, round face, short stature, brachydactyly (BD), subcutaneous ossifications and mental retardation. AHO features occur regardless of the parental origin of the Gsα mutation, because AHO features are thought to result from Gsα haploinsufficiency, primarily in those tissues where Gsα expression is biallelic. Consistent with this interpretation, changes in growth plate chondrocytes and subcutaneous ossifications occur, regardless of whether the disrupted allele is inherited from the mother or the father (20,21). Thus, AHO features are seen both in patients with maternal mutations i.e. PHP and paternal mutations, e.g. pseudo-PHP (pPHP), which is characterized by absence of PTH and/or hormonal resistance (Table 1). However, recent data from human studies have revealed that Gsα imprinting may be present in some features of AHO, that is obesity and cognitive impairment occur predominantly in patients with PHP (22,23).

### Pseudohypoparathyroidism Classification

PHP is subdivided into type I and type II. Type I is defined as the failure to increase both urinary cAMP and urinary phosphate excretion in response to exogenous PTH administration (1,2,3,4,5,24). In PHP-II, urinary cAMP generation in response to exogenous PTH administration is normal, but the urinary excretion of phosphate is impaired (25). Although the common biochemical features of PTH resistance are hypocalcemia, hyperphosphatemia, and elevated PTH levels, and found in PHP-Ia, PHP-Ic, and PHP-Ib; AHO is the part of clinical picture in PHP-Ia, PHP-Ic, pPHP and occasionally in PHP-Ib. In PHP-Ia/PHP-Ic, in addition to PTH resistance, hypothyroidism, growth hormone deficiency and hypogonadism are also demonstrable reflecting target-organ resistance to thyroid-stimulating hormone (TSH), growth hormone-releasing hormone (GHRH) and gonadotropins, respectively (1,2,3,4,5).

This complex classification of PHP is based on several distinct criteria, including the presence of AHO features, hormone resistance, urinary cAMP and phosphaturic response to exogenous PTH and Gsα activity (Table 1). However, there are some combinations of features which do not fit readily into this classification, especially with recent development in the field.

### Controversies in Pseudohypoparathyroidism Type I

The presence or absence of hormonal resistance is the one of the key findings, which differentiates PHP from pPHP, maternal from paternal mutations, respectively. However, mild resistance to PTH and possibly to other hormones such as TSH, has been described in patients carrying a paternal GNAS mutation, that is patients with pPHP (26), so that hormonal resistance is now not only associated with PHP, but with pPHP as well.

Another cornerstone of the earlier classification of PHP is presence or absence of features of AHO, which differentiates PHP-Ia/PHP-Ic from PHPI-b. However, a number of reports from the last decade have also shown that AHO features can exist in patients with epigenetic abnormalities of GNAS or namely PHP-Ib (27,28,29,30). Furthermore, GNAS methylation changes reminiscent of PHP-Ib have been reported in PHP-Ia patients with GNAS deletions (31). These findings suggest a molecular and clinical overlap between the two subtypes.

The measurement of Gsα protein activity from erythrocyte membranes is one diagnostic method used for differentiating PHP-Ic from PHP-Ia/pPHP, in patients with AHO features and carrying GNAS coding mutations. Additionally, according to the previous criteria, Gsα activity is expected to be normal in patients with PHP-Ib (1,2,3,4,5,6). However, recently PHP-Ib patients have been shown to have a moderate reduction in Gsα activity, in a similar but less severe manifestation as patients with PHP-Ia/pPHP (32). Thus, PHP-Ib patients having methylation abnormalities and with AHO features might also have low Gsα activity and the clinical and biochemical findings of these patients are consistent with PHP-Ia (32). On the other hand, if Gsα activity is normal in the patient with PHP-Ib and AHO features, the patients could be described as PHP-Ic, clinically and biochemically (27,28,29,30,32).

Additionally, molecular defects are not unique to PHP-Ic. The loss-of-function mutations in the carboxyl-terminus of GNAS, causing disruption of receptor-mediated activation but conservation of adenylyl cyclase receptor-independent activation, lead to PHP-Ic (33,34,35). And methylation defects, as found in PHP-Ib could be another molecular defect present in patients described clinically and biochemically as PHP-Ic (34).

There are too many inconsistencies described in the literature of PHP-I subtypes, both clinically, genetically and biochemically when using the earlier classification so that a newer, comprehensive classification would be welcome.

Furthermore, progressive osseous heteroplasia (POH) is a distinct entity described in patients with paternally inherited GNAS mutations, usually causing truncation of the gene product (36). Features typical of AHO and hormone resistance have been detected in some patients with POH. Conversely, some PHP-Ia patients with maternal mutations present with POH-like progressive deepening of the heterotopic ossifications (37,38). Furthermore, POH lesions show a mosaic distribution and follow dermomyotomes, usually with a unilateral pattern. Experimental evidence has shown that a loss of heterozygosity at the GNAS locus, with somatic mutations in a progenitor cell of somitic origin, may cause severe, progressive heterotopic ossifications that show a similar unilateral distribution (39).

### Controversies in Pseudohypoparathyroidism Type II

The differentiation of PHP-I from PHP-II is made by comparing the in vivo response to exogenous PTH in terms of nephrogenic cAMP synthesis and phosphaturia. The presence of cAMP elevation without phosphaturia marks PHP-II (24,25). Until 2011 no clear etiopathogenesis had been described for PHP-II (40). However, then and since, patients with acrodysostosis, have been found to exhibit biochemical abnormalities found in PHP-II. In addition, heterozygous mutations in PRKAR1A, which encodes the regulatory subunit of protein kinase A (PKA) and PDE4D, which encodes phosphodiesterase type 4, have been found in patients with acrodysostosis (40,41,42). Both PRKAR1A and PDE4D have a role in cAMP generation, down stream of Gsα. Thus, a heterogeneous group of rare diseases, characterized by skeletal dysplasia, has been included in the classification of PHP.

Acrodysostosis is characterized by skeletal dysplasia and has characteristic features, including BD, facial dysmorphism and, in some cases, mental retardation (43,44,45,46,47). Hormone resistances, usually PTH and/or TSH resistance, have been detected in about 60-70% of acrodysostosis patients with a PRKAR1A mutation and in 10-20% of cases with PDE4D mutations. However, typical facial features and more generalized BD distinguishes acrodysostosis from PHP (46,48). On the other hand, it has been shown that some cases with a phenotype typical of PHP-Ia also have PRKAR1A mutations (49,50).

Another disease that has been shown to involve the cAMP pathway is hypertension and brachydactyly syndrome (HTNB-Bilginturan syndrome, OMIM #112410) which is characterized by hypertension, BD type E (BDE) and short stature. Heterozygous mutations in PDE3A have been identified in patients affected with HTNB (51). Of note, BDE and short stature are clinical features of AHO.

Although these two diseases, acrodysostosis and HTNB syndrome exhibit molecular defects in the PTH-cAMP pathway and are clinically identical to PHP/pPHP, they were not previously included in the classification of PHP. Furthermore, disorders associated with an impaired function of PTH1R, i.e. Blomstrand and Eiken skeletal dysplasia, are also currently not included in the classification of PHP. In addition, other diseases featuring defects in cAMP and its downstream pathway, should have a place in the classification if they are described in the future.

### Rationale for the New Classification

In light of this new evidence the EuroPHP network, which is composed of experts from different independent centres, proposed a new classification to create a uniform terminology and classification based on the current knowledge of PHP (52). The term “inactivating PTH/PTHrP signalling disorder” (iPPSD) was selected since it describes the common mechanism responsible for the diseases, encompasses all disorders related to this pathway and was flexible enough to incorporate new development in this field (52).

The terms “PHP” and “pPHP” are confusing, both for description of the diseases and for use in communication. iPPSD is more compact and describes a group of disorders which makes the disease classification easier from the beginning. For the diagnosis of iPPSD, major and minor criteria have been described and a minimum of one of the major criteria is mandatory for clinical diagnosis of iPPSD (see Table 2) (52). PTH resistance or ectopic ossifications could be diagnostic for iPPSD with or without the presence of minor criteria. However, since BDE is a common feature of several other diseases and syndromes, in patients exhibiting BDE at least one major or two minor criteria should also be present for a diagnosis of iPPSD.

The entities included in iPPSD classification, with known molecular causes of impaired PTH/parathyroid hormone-related protein (PTHrP) signaling (52) are:

- Inactivating mutations of PTH1R

- Heterozygous inactivating mutations in the coding sequence of GNAS-Gsα

- Methylation changes of the DMRs of GNAS caused by deletions or duplications (STX16; NESP; GNAS-AS1) or paternal UPD of chromosome 20q or unknown mechanism(s)

- Heterozygous mutations of PRKAR1A leading to reduced PKA activity

- Heterozygous activating mutations of PDE4D

- Heterozygous activating mutations of PDE3A

### Major and Minor Criteria

### Major Criteria

**1. PTH resistance:** PTH resistance is defined as elevated PTH with or without hypocalcemia, hyperphosphatemia. Resistance occurs only at the renal proximal tubule and distal renal tubule and PTH is functionally intact and therefore, the patients will have hypocalciuria (1,2,3,4,5).

For evaluation of PTH resistance and to differentiate PTH resistance from normocalcaemic hyperparathyroidism, renal failure, vitamin D deficiency and any form of secondary hyperparathyroidism, the following laboratory tests should be performed; ionized calcium, total calcium, phosphate, magnesium, PTH, vitamin D (25-hydroxyvitamin D), creatinine, urinary calcium and urinary phosphate excretion. A PTH infusion test is reserved for challenging cases (1,2,3,4,5,52).

**2. Ectopic ossification:** Ectopic ossifications are foci of bone formation in the adipose or dermal tissue, which manifest as superficial, subcutaneous nodules (1,2,3,4,5). Progression of heterotopic osseous calcifications, usually from the dermal and subcutaneous tissues to the deeper tissues, such as muscles and tendons may be seen and defined as POH (36,37,38). In children, ectopic ossifications are highly suggestive of an inactivating GNAS mutation, i.e. iPPSD (52).

Diagnosis of ectopic calcification can be made by inspection and palpation on physical examination and may be detected by X-ray imaging if tissue is large enough. In selected cases, diagnosis may involve biopsy, but it is not recommended due to an increased risk for progression of biopsied osseous tissue (52). Fibrodysplasia ossificans progressiva (OMIM #135100) and post-traumatic osteoma cutis should be differentiated (53). Calcification rather than ossification should be considered as a differential diagnosis, as in tumoral calcinosis which is related to the defective activity of fibroblast growth factor 23 (FGF23), in which mutations in FGF23, GALNT3 and α-klotho have been identified (54).

**3. BDE:** BD refers to shortening of the fingers, toes or both. BD in iPPSD should be classified as BDE (OMIM #113300), which is characterized by variable shortening of the metacarpals, with more or less normal length of phalanges, occasionally accompanying shortened metatarsals (55). Hypoplastic and partially fused metacarpal epiphyses, seen on radiographs, are the cause of BD and lead to BDE. In addition, the terminal phalanges are often short (55). It can either present in isolation or as part of a genetic disorder, most of which are included in the iPPSD classification (56).

Almost all patients with GNAS mutations have BD and decreased Gsα activity, which is usually decreased by around 50% (57,58,59). Although, Gsα activity is supposed to be normal in cases with methylation abnormalities such as in the entity known as PHP-Ib formerly, PHP-Ib patients with an AHO phenotype have more severely diminished Gsα activity levels than those who do not have the AHO phenotype (32). Furthermore, BD has been detected in both patients with a genetic mutation and in those with an imprinting error in PHP-Ib but at differing median ages of detection; 7.2 years in the former and 13.2 years in the latter (60). These results could be related to the degree of the Gsα functional impairment with a more severe loss of function leading to earlier BD development. It can be difficult to detect BD, especially in early childhood, and tends to become more evident during early puberty. BD can be overlooked when all bones are short as in acrodysostosis which has affected the patient since early childhood (61).

Clinical and radiological evaluation of hand bones are necessary for a diagnosis of BDE. On clinical examination, by using a straight ruler at the head of the metacarpals of the closed fist, the tips of 3^rd^, 4^th^ and 5^th^ metacarpals should be in a line and touching the ruler. If the 4^th^ or 5^th^ metacarpals are receding, this can be accepted as a positive metacarpal sign, also known as Archibald’s sign (55,62,63). The evaluation on X-rays can be done in a similar fashion (55,63). However, normally this sign is positive in only 9.6% of individuals and if a deviation of more than 2 mm is accepted as a limit, only 0.5% of individuals have the sign (64). In addition, if all bones are short, this metacarpal sign will be negative. If so, each metacarpal and phalangeal bone should be measured and evaluated separately (metacarpo-phalangeal profile). If shorter than 2 standard deviation scores (SDS) for the individual bone, it is accepted as short and BD (65). Differential diagnoses for BDE are Turner syndrome, tricho-rhino-phalangeal syndrome (TRPS) including TRPS type I, (OMIM #190350), TRPS type II (OMIM #150230) and TRPS type III, (OMIM #190351), BDE with short stature, parathyroid hormone-like hormone (PTHLH, OMIM #613382), isolated BDE: HOXD13 type (OMIM #113300) and BD mental retardation syndrome (OMIM #600430) (56).

While existence of PTH resistance or ectopic ossifications are considered diagnostic for iPPSD as major criteria; BD is less specific and should, therefore, be present with at least one other major or two minor criteria to consider the diagnosis of iPPSD.

### Minor Criteria

**1. Thyroid-Stimulating Hormone Resistance**

TSH resistance is usually characterized by mildly elevated TSH levels with a normal or low-normal free thyroxine (T4) level. TSH levels are usually below 50 mIU/L (66,67). Sometimes patients present with clinical symptoms of hypothyroidism, such as prolonged jaundice, macroglossia, hypothermia and umbilical hernia in neonates or constipation and listlessness in infants (66,68).

Hypothyroidism occurs in the absence of goiter and markers of autoimmune disease (66,67). In laboratory evaluation, TSH, free-T4, anti-thyroid antibodies and thyroid ultrasound should be performed. TSH receptor inactivation mutation can be considered in the differential diagnosis (52,66,67).

TSH resistance could be a first manifestation of iPPSD, especially if referred from the neonatal screening program for congenital hypothyroidism (68,69).

**2. Other Hormone Resistances**

Other hormone resistances are also present in iPPSD. Growth hormone deficiency due to resistance to GHRH, is the next most frequent resistance reported, and found in 60% of patients with PHP-Ia (70,71,72). Calcitonin resistance has also been also described in patients with PHP-Ia, but with no known associated clinical or biochemical abnormalities (67). Gonadotropin resistance, with elevated follicle-stimulating hormone (FSH) and luteinizing hormone (LH) levels, is a further G-protein coupled hormone resistance reported in iPPSD (73,74). Glucagon and adrenaline resistances have been demonstrated through in vivo testing in patients with low Gsα bioactivity (75,76).

For evaluation of growth hormone deficiency; insulin-like growth factor (IGF)-1, IGFB-3 and growth hormone stimulation tests can be performed, if necessary. Serum measurements of calcitonin, LH and FSH are helpful if the respective resistance is suspected and in addition a gonadotropin-releasing hormone/LH-releasing hormone test may be performed.

### Motor and Cognitive Retardation or Impairment

Psychomotor and cognitive impairments have been described as a feature of AHO. A significant proportion of patients (40-70%) with a maternal coding mutation of GNAS, (formerly PHP-Ia) has been shown to have cognitive impairment (22,77). However, cognitive impairment is seen rarely in patients with paternally inherited GNAS mutations (PPHP, POH) ranging from 0% to 10% of cases (78). The patients with methylation abnormalities, i.e. PHP-Ib, may also have cognitive impairment (79,80,81) especially if they have AHO features, as cognitive impairment is reported in almost half of them (30). Additionally, varying severity of psychomotor and cognitive impairment has been described in some patients with acrodysostosis (42,44,45). It has been suggested that psychiatric disorders may be part of the disease spectrum (82). However, patients with paternal mutations of GNAS or epigenetic modifications of GNAS DMRs seem to be unaffected (22,83).

### Intrauterine and Postnatal Growth Retardation

Intrauterine growth retardation (IUGR) has been frequently observed in patients with inactivating GNAS coding mutations. Although both paternal and maternal inherited mutations are associated with IUGR, patients harbouring mutations on the paternal GNAS allele are more severely affected, especially when the mutation is in exons 2 to 13, compared with patients with GNAS exon 1/intron 1 mutations (84). The reason for paternal GNAS exon 2-13 mutations causing more severe IUGR is due to an impairment of another transcript of GNAS, XLαs, which is essential for early postnatal adaptation to feeding and survival, as well as glucose counterregulation (85,86). IUGR has also been described in other iPPSD, such as acrodysostosis with mutations in PRKAR1A or PDE4D, and in patients with mutations in PDE3A (40,41,49,51). However, loss of methylation at the maternal GNAS A/B: PHP-1b has been associated with increased intrauterine growth and high birth weight (87).

Postnatal growth retardation resulting in short final height is a common finding in PHP-Ia and acrodysostosis. Growth hormone deficiency and premature closure of the epiphysis are the causes of short stature (40,41,70,88). Rarely, growth retardation has also been described in PHP-Ib (27,30) and in patients with Eiken dysplasia (89).

### Obesity/Overweight

Obesity or overweight is commonly present but, is possibly the most nonspecific minor sign of iPPSD. However, early onset obesity is an important clinical feature manifesting from the first few months of life and resulting in severe obesity during infancy. However, obesity tends to improve as the patient ages. In adulthood, only about two thirds of PHP-Ia are obese with a mean body mass index (BMI) Z-score of 1.7±0.2 (77,90,91).

Patients with maternally inherited GNAS coding exon mutations, but not those carrying mutations on the paternal allele, have obesity/overweight. This may be helpful in differentiating PHP-Ia from pPHP. Growth hormone deficiency, impaired lipolytic response to adrenaline (76) or decreased resting energy expenditure (92) may all contribute to the development of obesity in patients with mutations on the maternal allele (23,91). Obesity is also a frequent feature in patients affected with acrodysostosis (40,49,93). For evaluation, weight charts and BMI SDS or percentile charts are necessary. Monogenic obesity stemming from leptin/melanocortin pathway abnormalities should be considered in differential diagnosis of early onset obesity (94).

### Flat Nasal Bridge and/or Maxillar Hypoplasia and/or Round Face

Patients with acrodysostosis have typical facial features with flat nasal bridge and/or maxillar hypoplasia and patients with PHP-Ia have a round face which is inconsistent with the degree of obesity. These findings are, however, nonspecific (19,45).

### The New Classification (Figure 1)

The former classification of PHP/pPHP is based on the clinical and biochemical phenotype. However, a new classification, iPPSD, has been identified according to described clinical and biochemically criteria. Further subtyping will be possible by identifying the underlying molecular genetic or epigenetic defect. Thus, the term iPPSD refers to the pathophysiology, which is impairment of PTH/PTHrP signaling, and the number refers to the underlying molecular defect as shown below (52).

### The Classification of Inactivating PTH/PTHrP Signalling Disorder (52)

iPPSD: Clinical/biochemical diagnosis based on the major/minor criteria described, without any genetic investigation/diagnosis.

iPPSD1: Loss-of-function mutation in PTH1R.

iPPSD2: Loss-of-function mutation in Gsα.

iPPSD3: Methylation change(s) at one or more GNAS DMRs, associated with or without a genetic deletion (STX16, NESP55, AS etc.) or cytogenetic (UPD) defect. The loss of methylation at the GNAS A/B is the common mechanism shared by these patients.

iPPSD4: Mutation in PRKAR1A leading reduced PKA activity.

iPPSD5: Gain-of-function mutation in PDE4D mutation.

iPPSD6: Gain-of-function mutation in PDE3A mutation.

iPPSDx: Absence of any genetic/epigenetic defect after molecular investigations of known genes described above but fitting the criteria for iPPSD.

iPPSDn+1: Identification of a new gene and/or molecular defect will increment the number of iPPSD types by one, i.e. iPPSD7, iPPSD8 and so on.

With this new classification, the disorders were stratified according to etiopathogenesis, thus mechanism and simplified the concept of the overlapping disorders under a single umbrella. Additionally, it is flexible enough to accommodate new defects which may be discovered in the future. However, with this classification, the parental origin of the genetic/epigenetic defect is not taken into account, although iPPSD2 and iPPSD3 are imprinting disorders and their clinical presentation depends on the parental origin of inheritance. Although multiple hormone resistance, including PTH resistance, are largely associated with maternal GNAS mutations and isolated AHO and/or POH are more often associated with paternal GNAS mutations, hormone resistance and POH may be seen in both maternal and paternal inactivating GNAS mutations. Therefore, the new classification does not include parental origin of mutation but for genetic counseling this point should be considered. The mechanism of the two allelic GNAS mutations can be considered alike. Another point of this classification is the inability to sub-classify individuals with purely clinical findings-molecular analysis is mandatory. Cases should be classified as iPPSD, not iPPSDx, pending definitive molecular diagnosis.

Furthermore, PTHR1 has been included in the classification. However, two main ligands of PTHR1, PTH and PTHrP and related disorders are not chosen as a part of classification. Since, BDE with short stature seen in patients with PTHLH mutations, encoding PTHrP, (95,96), this point could be argued. Since these disorders are not primarily related to the signaling pathway defect, it is not included in the definition of main classification.

## CONCLUSION

A new classification has been established by the EuroPHP network to cover all disorders of the PTH receptor and its signaling pathway. iPPSD is the new name proposed for this group of conditions and which are further divided into the subtypes from iPPSD1 to iPPSD6. With this new classification, it is aimed to clarify the border of each different subtype of disease and make the classification according to molecular pathology. The iPPSD group is a growing group of conditions and new entities can readily be fitted into this classification.

## Figures and Tables

**Table 1:**
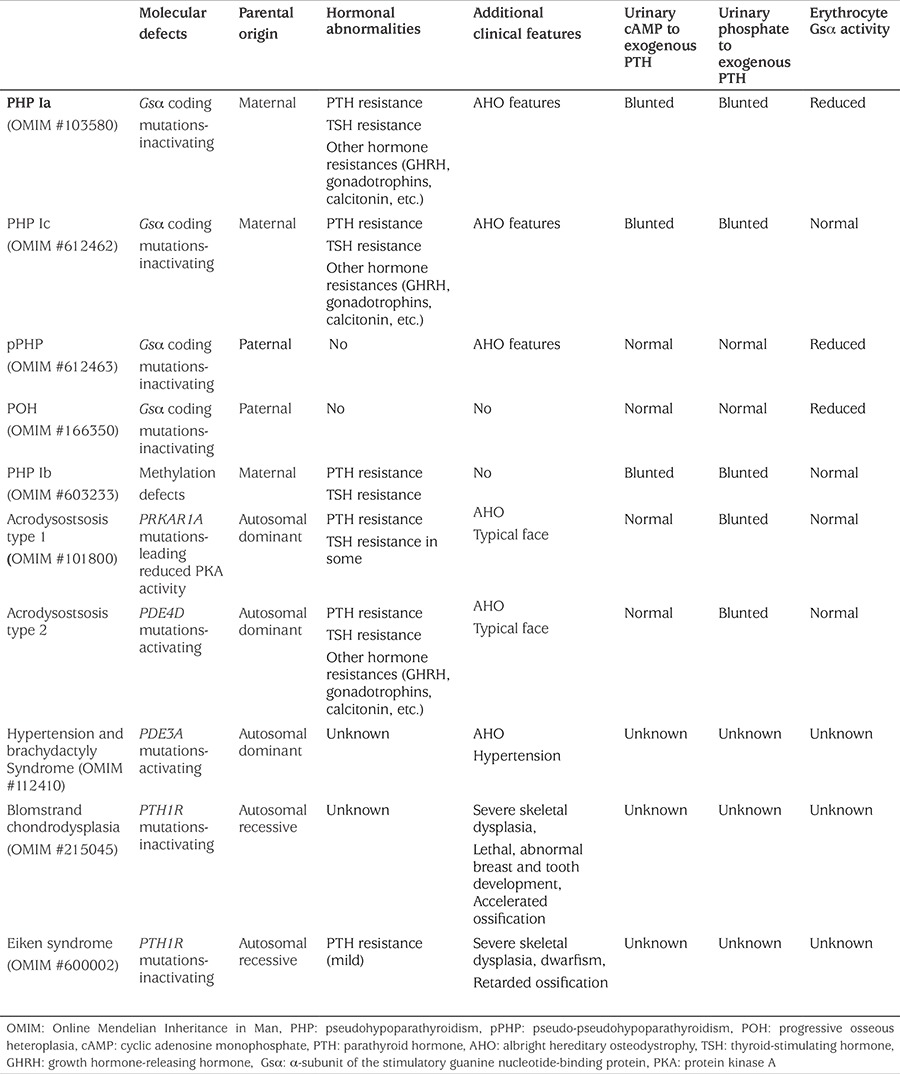
Disease related parathyroid hormone/parathyroid hormone-related protein and cyclic adenosine monophosphate signaling pathway and former classification according to clinical features and molecular defects

**Table 2:**
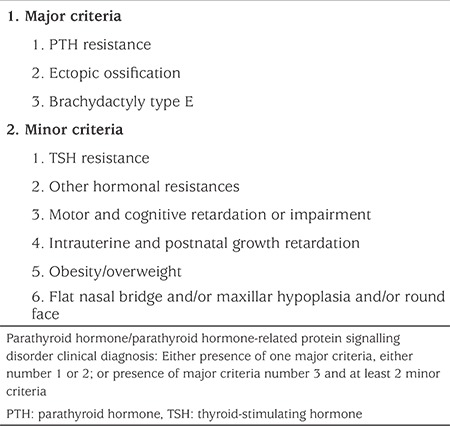
Diagnosis of inactivating parathyroid hormone/parathyroid hormone-related protein signalling disorder with major and minor criteria

**Figure 1:**
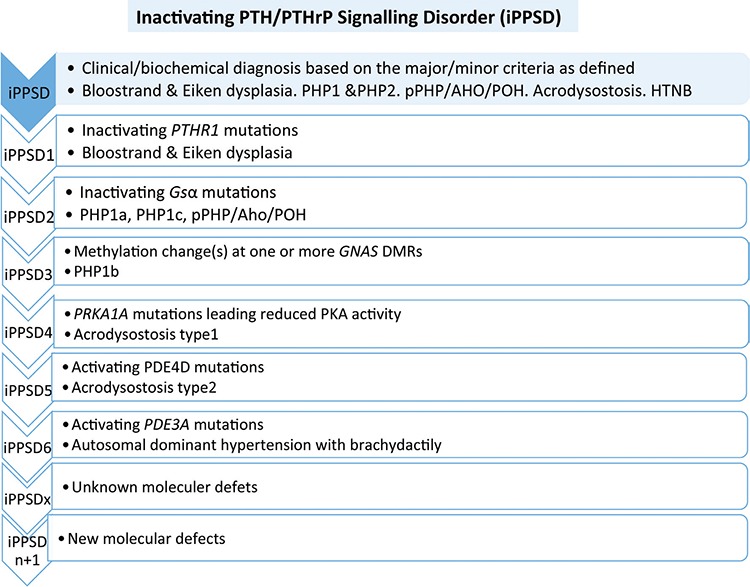
The new classification proposed by the European Pseudohypoparathyroidism Network (52) with new nomenclature on the left with molecular defects and the disease names listed in the right column PTH: parathyroid hormone, PTHrP: parathyroid hormone-related protein, iPPSD: inactivating parathyroid hormone/parathyroid hormone-related protein signaling disorder, DMRs: differentially methylated regions, POH: progressive osseous heteroplasia, PHP: pseudohypoparathyroidism, pPHP: pseudopseudohypoparathyroidism, AHO: Albright hereditary osteodystrophy, PKA: protein kinase A, HTNB: hypertension and brachydactyly syndrome
